# Multifractal Multiscale Analysis of Human Movements during Cognitive Tasks

**DOI:** 10.3390/e26020148

**Published:** 2024-02-08

**Authors:** Andrea Faini, Laurent M. Arsac, Veronique Deschodt-Arsac, Paolo Castiglioni

**Affiliations:** 1 Department of Cardiovascular, Neural and Metabolic Sciences, Istituto Auxologico Italiano, IRCCS, 20149 Milan, Italy; a.faini@auxologico.it; 2 Department of Electronics Information and Bioengineering, Politecnico di Milano, 20156 Milan, Italy; 3 University of Bordeaux, CNRS, Laboratoire IMS, UMR 5218, 33405 Talence, France; laurent.arsac@u-bordeaux.fr (L.M.A.); veronique.arsac@u-bordeaux.fr (V.D.-A.); 4 Department of Biotechnology and Life Sciences (DBSV), University of Insubria, 21100 Varese, Italy; 5 IRCCS Fondazione Don Carlo Gnocchi ONLUS, 20148 Milan, Italy

**Keywords:** detrended fluctuation analysis, multifractal, multiscale analysis, cycling, Tetris, multifractal cumulative function, Legendre spectrum

## Abstract

Continuous adaptations of the movement system to changing environments or task demands rely on superposed fractal processes exhibiting power laws, that is, multifractality. The estimators of the multifractal spectrum potentially reflect the adaptive use of perception, cognition, and action. To observe time-specific behavior in multifractal dynamics, a multiscale multifractal analysis based on DFA (MFMS-DFA) has been recently proposed and applied to cardiovascular dynamics. Here we aimed at evaluating whether MFMS-DFA allows identifying multiscale structures in the dynamics of human movements. Thirty-six (12 females) participants pedaled freely, after a metronomic initiation of the cadence at 60 rpm, against a light workload for 10 min: in reference to cycling (C), cycling while playing “Tetris” on a computer, alone (CT) or collaboratively (CTC) with another pedaling participant. Pedal revolution periods (PRP) series were examined with MFMS-DFA and compared to linearized surrogates, which attested to a presence of multifractality at almost all scales. A marked alteration in multifractality when playing Tetris was evidenced at two scales, τ ≈ 16 and τ ≈ 64 s, yet less marked at τ ≈ 16 s when playing collaboratively. Playing Tetris in collaboration attenuated these alterations, especially in the best Tetris players. This observation suggests the high sensitivity to cognitive demand of MFMS-DFA estimators, extending to the assessment of skill/demand interplay from individual behavior. So, by identifying scale-dependent multifractal structures in movement dynamics, MFMS-DFA has obvious potential for examining brain-movement coordinative structures, likely with sufficient sensitivity to find echo in diagnosing disorders and monitoring the progress of diseases that affect cognition and movement control.

## 1. Introduction

The complex dynamics of human movements result from the interactions among perceptive, cognitive, and motor systems aimed at responding to changes in the environment or achieving specific tasks. Such interactions are responsible for continuous motor adaptations, and the resulting variability reflects the superposition of fractal processes with different power laws. For this reason, the intrinsic variability of human movements is characterized by multifractal dynamics [1,2,3,4].

Among the estimators of the multifractal spectrum, the detrended fluctuation analysis (DFA) [5] for multifractal series (MF-DFA) [6] has gained popularity in the study of motor control due to the statistical performance of the recently proposed focus-based approach [7]. In fact, a limit of most studies is to find a reasonable trade-off between two contrasting requirements: On the one hand, recording long time series to improve the statistical consistency of the regressions that estimate the scale exponents; on the other hand, restricting the duration of motor or cognitive tasks to avoid fatigue, learning or habituation phenomena. The focus-based approach assumes that the DFA regressions of each multifractal exponent converge to a theoretical focus, and it improves the estimates by calculating the focus empirically and by forcing the passage of the regression lines through the calculated focus. Since it provides statistically stable estimates for relatively short series, the focus-based MF-DFA has been used to assess human movements during concurrent visuomotor tasks [8] or complexity-matching phenomena between coupled neural networks [9].

Often, however, the fractal dynamics of physiological systems exhibit different power laws at different temporal scales, that is, a multiscale spectrum. This was soon recognized in the heart-rate series for which the DFA coefficients are traditionally estimated at short and long scales separately [5]. Multiscale analyses have also been used for the monofractal DFA of human movements [10,11]. By contrast, the focus-based approach implicitly assumes that the multifractal exponents are the same at all scales; thus, it cannot separate “short-term” from “long-term” exponents. Therefore, the possible presence of multiscale structures can only be inferred by progressively removing the shorter scales from the multifractal analysis [9].

In this regard, a method for quantifying the multifractal DFA coefficients at each scale separately has been proposed recently [12]. This method improves the statistical consistency of the estimates by splitting the series into maximally overlapped blocks and does not require any assumption on the linear convergence of the DFA variability functions into focus. Based on this approach, a scale-by-scale measure of multifractality that avoids the inapplicability of the Legendre transform when the convexity hypothesis is not satisfied has been also proposed [13]. This multifractal and multiscale approach (MFMS-DFA) has been applied so far to cardiovascular series only [14], but it could be a valuable tool for studying the temporal structures of the multifractal spectrum of human movements as well. By investigating the multifractal dynamics at different scales, such a tool could allow for better assessing the interactions of cognitive- and motor-neural networks in patients after a trauma, or more accurately monitoring the progression of degenerative diseases, as well as the effectiveness of rehabilitation treatments, over time.

Therefore, the present methodological work aims to evaluate whether the recently proposed MFMS-DFA allows effective identification of multiscale structures also in the multifractal dynamics of human movements and quantifying their possible alterations. For this aim, we will describe the multifractal multiscale structure of cycling by maintaining a target pedal rate as the reference multifractal motor task. Furthermore, we will quantify the possible alterations in the multifractal structure of the pedal revolution intervals when a cognitive task is performed simultaneously. We expect that the performance of the reference motor task may be altered due to the greater dual-task cognitive demand and because of the limitations in simultaneously processing the associated interfering streams of information [15,16]. Thus, we aim to describe the intrinsic multifractal dynamics that should characterize the time series of pedal revolution periods and the possible alterations induced in such structure by a dual-task cognitive load, which in our study consists of playing Tetris while cycling.

Therefore, if the recently proposed MFMS-DFA can properly assess the complex dynamics of human movements, we expect to identify possible alterations induced by the cognitive tasks in the multifractal structure at specific scales; and that the presence of multifractality, which is expected to characterize the intrinsic variability of human movements, should be detected also within this temporal structure of multifractal scale coefficients.

## 2. Materials and Methods

### 2.1. Subjects and Data Collection

Thirty-six healthy participants (12 females) aged 31.1 ± 12.5 years, all students or university members, gave their written informed consent to participate in this study, which was approved and authorized by the Institutional Review Board Faculte des STAPS and followed the rules of the Declaration of Helsinki.

In order to get temporal variability in movement repetitions emitted by a free-running neurophysiological system, the participants followed a synchronization-continuation paradigm [17]. They were asked to pedal on a friction-loaded cycle ergometer (Monark 818E, Monark, Vansbro, Sweden), imposing the cadence of 60 revolutions per minute (rpm) by a metronome for the first 60 s period (no recordings). The metronome was subsequently stopped, and the participants were asked to keep the same pedaling cadence for the next 10 min against a friction load amounting to 10N. A previous study already reported that such a cycling protocol generates fractal fluctuations in the pedaling cadence [18].

The duration of each pedal revolution period (PRP) was obtained thanks to a Light Meter Pod connected to a PowerLab acquisition system (ADInstruments, Sydney, Australia) at a 1 kHz sampling rate, detecting the changes in light when the pedal passed by the sensor. Each participant reiterated the above procedure in three conditions. The first is the reference cycling (C) condition with the participant’s arm resting on an elevated table in front of the bicycle. The second is the dual-task condition, cycling and playing “Tetris” [19] on a computer placed on the table (CT). In Tetris, the player observes a field on the screen in which pieces of different geometric shapes descend from the top. The player can rotate and move the pieces laterally and accelerate them as they fall to create the greatest number of complete horizontal lines of blocks; when a line is completed, it disappears granting points. Thus, Tetris can be considered a progressively demanding cognitive task requiring visuospatial functions. The third condition is again a dual-task performance but cycling simultaneously playing Tetris collaboratively (CTC) on shared screens with the one cycling concomitantly on their side (hidden by a board). In the latter condition, one subject could turn the pieces, the other one could shift them horizontally while they drop and accelerate the drop. Since in CTC the tasks of turning the pieces and moving them horizontally/vertically are no longer performed by a single player, as in CT, but each of the two players takes care of a single task, we expect a somehow lower cognitive load of playing Tetris collaboratively. Tetris collaborations were matched by gender and age.

**Figure 1:**
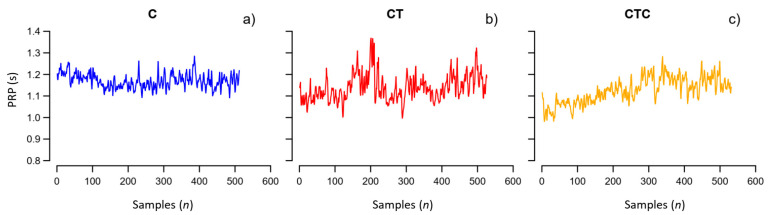
Example of pedal revolution periods, PRP. Recording in the same participant (**a**) during cycling, C; (**b**) cycling playing Tetris alone, CT; (**c**) cycling playing Tetris collaboratively, CTC.

We recorded the Tetris best score achieved by each participant during CT. At the end of each condition, participants reported the workload they perceived by compiling the NASA Task Load Index (NASA-TLX) questionnaire [20]. The score of the overall workload may range between the minimum value of 0 up to the maximum value equal to 100. Figure 1 shows an example of a PRP series recorded in one participant.

### 2.2. Multifractal Multiscale DFA

#### 2.2.1. Estimation of Multifractal Multiscale Coefficients

The PRP fluctuations around the desired set point of 60 rpm result from the superimposition and interaction of several factors. If these factors act independently without any underlying feedback control, we may expect that they produce uncorrelated deviations from the set point. On the other hand, if they interact with the higher brain center of the motor control to maintain the set point, we may expect long-term correlations in the PRP deviations. The DFA coefficients may characterize this phenomenon as being equal to 0.5 for uncorrelated fluctuations (like white noise) and 1.0 for a purely self-similar process like the 1/f noise. Thus, a decrease in the DFA coefficient might reflect a lower motor control of the higher brain center. In our fractal model of motor control, the multifractal analysis allows to separately describe interacting factors with different fractal dimensions; and the multiscale analysis might reveal shifts of the correlated fluctuations among the temporal scales, possibly providing further clues on the motor adaptation strategies during a dual-task performance.

We estimated the multifractal multiscale structure of the PRP series by the MFMS-DFA algorithm described and downloadable in [12]. Briefly, we calculated the cumulative sum, *y_i_*, of each PRP series and split *y_i_* into *M* maximally overlapped blocks of *n* samples. Then we detrended each block with a least-square polynomial regression and calculated the variance of the residuals in each *k-th* block, σ^2^*_n_*(*k*). The variability function *F_q_*(*n*) is the *q*-th moment of σ^2^*_n_* [6]:

(1) $$\left\{\begin{array}{c} F_{q}(n)={\left(\frac{1}{M}{\sum_{k=1}^{M}\left(\sigma_{n}^{2}\left(k\right)\right)}^{q/2}\right)}^{1/q}\;\mathrm{for}\;q\neq 0\\ \;F_{q}(n)=e^{\frac{1}{2M}\sum_{k=1}^{M}\ln\left(\sigma_{n}^{2}\left(k\right)\right)}\;\;\;\mathrm{for}\;q=0 \end{array}\right.$$

**Figure 2:**
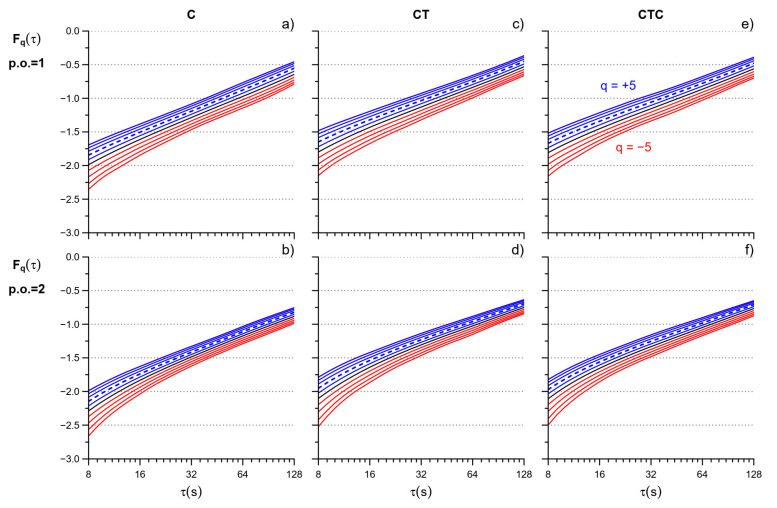
MF-DFA variability functions. F_q_(τ) after polynomial detrending of order 1 (p.o. = 1) in C (**a**), CT (**b**), and CTC (**c**) and order 2 (p.o. = 2) in C (**d**), CT (**e**), and CTC (**f**): average of 36 participants. F_q_(τ) in red for q < 0, blue for q > 0, and black for q = 0; the dashed line is q = 2 (moment order of the monofractal DFA). Note the larger effects of overfitting at the shorter scales for the second-order polynomial, which, however, is expected to better remove trends at the larger scales.

We calculated *F_q_*(*n*) for −5≤ *q* ≤ 5 and 6 ≤ *n* ≤ *L*/4, with *L* the length in samples of the PRP series. We mapped the scale units from number of pedal revolutions, *n*, to time τ, in seconds, with the transformation:

τ = *n* × μ_PRP_ (2)

where μ_PRP_ is the mean PRP, in seconds. We spline-interpolated the log τ axis evenly [21] between 8 and 128 s to consider the same scales in all the participants. Scales τ < 8 s were excluded to avoid the large estimation bias for negative *q* at shorter scales [12]. We calculated *F_q_*(τ) twice, for detrending polynomials of first and second order (Figure 2) because the second-order polynomial removes the longer trends more efficiently but it over-fits block sizes shorter than 12 beats [12,22,23].

We calculated the multifractal coefficients as the derivative of log *F_q_*(τ) vs. log τ separately for the two detrending orders. The final estimate, α(*q*,τ), combined the coefficients of the two detrending orders with a weighted average that progressively weights order 1 more at the shorter than longer scales, as described in [12]. To check the influence of possibly present long-term drifts in the recorded series, we recalculated α(*q*,τ) twice, after removing a linear or a quadratic drift from the original PRP series. We did not find substantial effects of drift removal when the scale coefficients were estimated by combining the two detrending orders (see Appendix A); thus, the analysis was performed without drift removal so as not to introduce an additional pre-elaboration stage.

#### 2.2.2. Statistical Comparison with the Reference Condition

We compared the α(*q*,τ) coefficients in “CT” and “CTC” vs. the reference “C” by the Wilcoxon signed-rank test for each *q* and τ. In this way, we obtained the Wilcoxon V signed-rank statistics as a function of both the moment order and scale: V(*q*,τ). The significance of observing a given V(*q*,τ) value was represented by a color map. The map allows visualizing the regions of the *q*-τ space with the more significant differences with the reference condition.

To stratify the results by skill levels, the group was subdivided into tertiles according to the individual Tetris score, and the comparisons were repeated in each tertile separately.

### 2.3. Degree of Multifractality Scale by Scale

#### 2.3.1. Cumulative Multifractality Function

We quantified the degree of multifractality at each scale by the cumulative function of the squared increments of scale exponents, α_CF_(τ):

(3) $$\alpha_{CF}\left(\tau \right)=\sum_{q=-4}^{5}{\left[\alpha \left(q,\tau \right)-\alpha \left(q-1,\tau \right)\right]}^{2}$$

For empirical physiological time series, this index of multifractality is more stable than the width of the singularity spectrum based on the Legendre transform [13].

#### 2.3.2. Surrogate Data Analysis

We employed two generators of surrogate data [24]: (1) the “random phase” (RP) algorithm that preserves the second-order statistics (e.g., the power spectrum) but not the amplitude distribution by shuffling the phases of the Fourier spectrum; and (2) the “iterative amplitude adjusted Fourier transform” (IAAFT) algorithm, which tries to preserve both the power spectrum and the amplitude distribution through an iterative procedure of gaussianization, phase randomization, and de-gaussianization. We created 100 surrogate series for each PRP recording, both for the RP- and the IAAFT generator.

#### 2.3.3. Statistical Comparison with the Surrogate Data

We tested the presence of multifractality at each τ by comparison with the surrogate data. For each participant *j*, with 1 ≤ *j* ≤ 36, we first calculated the cumulative function of the original series “O”, $\alpha_{CF}^{O,j}(\tau )$. Then we calculated the cumulative function of each of 100 RP-surrogate series *i*, $\alpha_{CF}^{i,j}(\tau )$, with 1 ≤ *i* ≤ 100, and their median values, $\alpha_{CF}^{M,j}(\tau )$. Finally, we tested the presence of multifractality evaluating whether the cumulative function of the original series, $\alpha_{CF}^{O,j}(\tau )$, was statistically greater than the median cumulative function of the surrogate series, $\alpha_{CF}^{M,j}\left(\tau \right)$, at each scale τ, over the group of N = 36 participants, by the Wilcoxon one-tailed paired test. The test was repeated for the IAAFT-surrogate series.

All the statistics were conducted using R (R Core Team, 2023) and RStudio (Posit Team, 2023).

## 3. Results

### 3.1. MFMS-DFA and Cognitive Tasks

**Figure 3:**
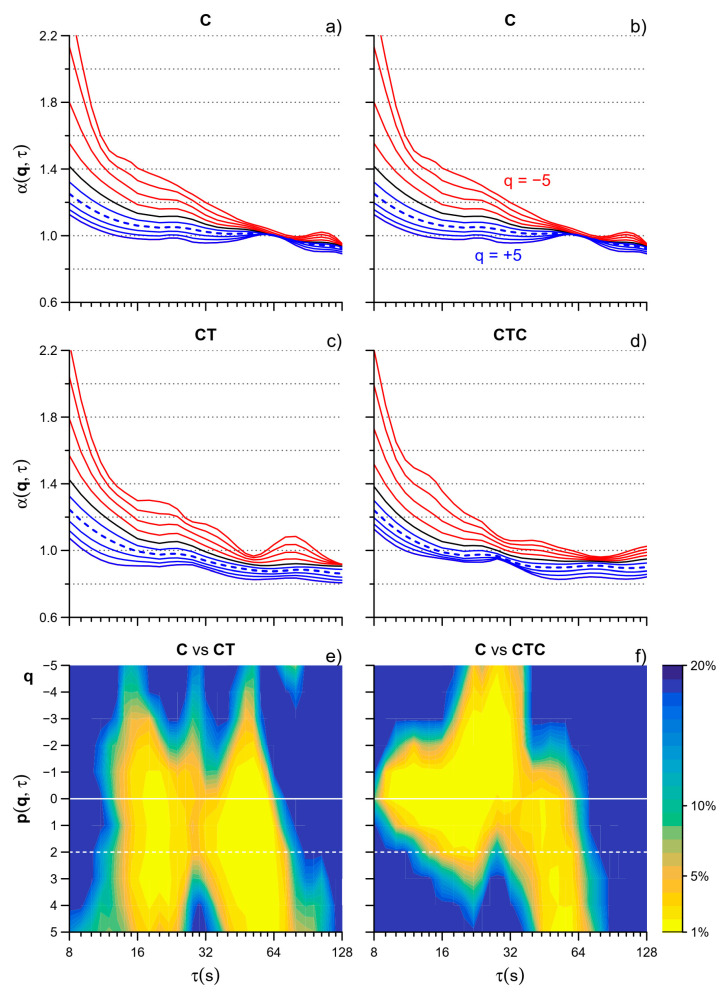
MFMS-DFA coefficients in C (**a**,**b**), CT (**c**), and CTC (**d**) and color maps of the statistical significance of the Wilcoxon p comparing C vs. CT (**e**) and CTC (**f**); α(q,τ) in red for q < 0, blue for q > 0, black for q = 0; dashed lines indicate q = 2 (moment order of the monofractal DFA).

Figure 3 compares the α(*q*,τ) coefficients in the reference cycling condition C with cycling playing Tetris alone, CT, or collaboratively, CTC. Color maps show the statistical significance at *p* < 1% in bright yellow and point out marked alterations induced by the cognitive tasks. Playing Tetris alone produces changes at two scales, around τ = 16 s and τ = 64 s, mainly for positive moment orders (the differences quickly lose significance for *q* < −1). The upper left panel shows that for *q* > 0, α(*q*,τ) decreases from C to CT. In particular, for *q* = 2 (moment order of the monofractal DFA, dashed lines in Figure 3), the scale coefficient at τ = 16 s decreases from the value of a fractional Brownian motion process (α > 1) to the value of pink noise (α = 1) and at τ = 64 s decreases from pink noise to the value of a fractional Gaussian noise (α < 0.8).

Playing Tetris collaboratively (CTC) produces marked changes too. While at τ = 64 s the color map of C vs. CTC highlights the same α decrease we found in the C vs. CT comparison, the decrease is less pronounced at τ = 16 s. Furthermore, playing Tetris collaboratively slightly but significantly decreases α around τ = 32 s for *q* < 0.

### 3.2. Multifractality

**Figure 4:**
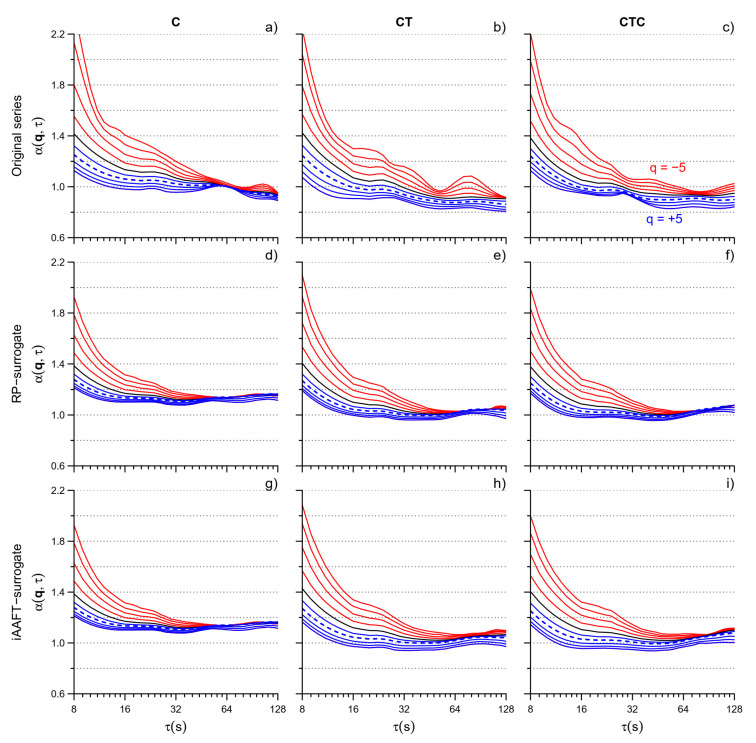
MFMS-DFA coefficients for original and surrogate series. α(q,τ) of the original series in C (**a**), CT (**b**), and CTC (**c**); RP-surrogate series in C (**d**), CT (**e**), and CTC (**f**); and IAAFT-surrogate series in C (**g**), CT (**h**), and CTC (**i**). For the original series, the figure shows the average over N = 36 participants; for the surrogate series, the median over 100 surrogates was calculated for each of the N = 36 participants and the figure shows the average over N = 36 medians.

Figure 4 compares the α(*q*,τ) functions for the original and surrogate series. Visually, a lower dispersion of the multifractal coefficients is apparent at all the scales τ.

**Figure 5:**
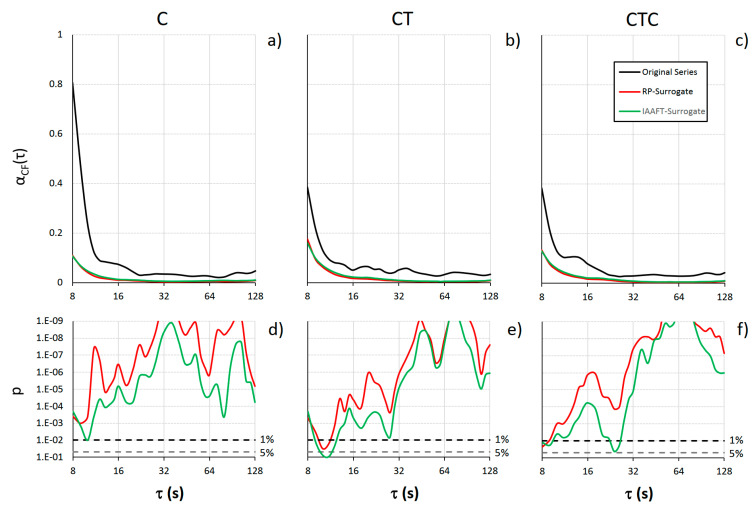
Multifractality cumulative function α_CF_(τ). Averages over 36 participants for the original series and its RP- and IAAFT surrogates in C (**a**), CT (**b**), and CTC (**c**). Statistical significance p of the difference between original and surrogate series in C (**d**), CT (**e**), and CTC (**f**): When p is above the dashed lines at 5% and 1% levels, α_CF_ is significantly lower for the surrogate than the original series at the 0.05 and 0.01 statistical significance.

The visual trend is confirmed by Figure 5 which shows the degree of multifractality of the original and surrogate series and their statistical comparison. The cumulative function α_CF_(τ) is significantly lower for the surrogate data over most of the scales, indicating an almost ubiquitous presence of multifractality at all τ’s. It is worth noting a greater significance of multifractality in C than in CT or CTC at τ < 32 s for both the RP and IAAFT surrogates and a systematically larger *p* (i.e., lower significance) when the comparison is performed against the IAAFT than the RP surrogate.

### 3.3. Stratification by Skill Level

Participants were subdivided into tertiles according to their Tetris best score. The I tertile included 12 participants with a best score between 1400 and 7900; the II tertile 12 participants with a best score greater than 7900 up to 10,967; and the III tertile the remaining 12 participants, with a best score from 10,967 to 32,200. Table 1 reports the general characteristics of participants in each tertile separately. The three subgroups had similar age and sex composition. They also reported similar scores for the perceived workload of the three tasks, with greater scores for the dual tasks.

**Figure 6:**
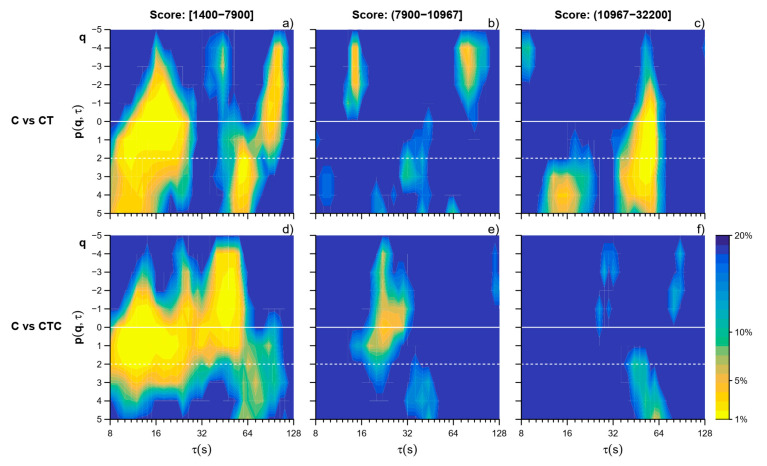
Wilcoxon p significance of the comparison of the MFMS-DFA coefficients between the reference C vs. CT and CTC by tertile of Tetris score. Color codes as in Figure 3 compare C vs. CT in the I (**a**), II (**b**), and III (**c**) tertile and C vs. CTC in the I (**d**), II (**e**), and III (**f**) tertile.

The effects of playing Tetris, alone or collaboratively, are evident in the I tertile (participants with the lower skill): By contrast, almost no effects of playing Tetris collaboratively appear in the tertile with the highest skill level (Figure 6). The same high-skill participants, however, when they played Tetris alone, that is, in the experimental condition where they reached their best scores, showed a clear alteration in the α(*q*,τ) coefficients (CT vs. C) at the scales around τ = 64 s and *q* > 0.

## 4. Discussion and Conclusions

This work applied the recently proposed MFMS-DFA to quantify for the first time the scale-by-scale profile of the multifractal exponents of motor time series. Multiscale DFA is common in studies on cardiovascular dynamics, and the MFMS-DFA has been already used on heart rate and blood pressure beat-to-beat recordings. By contrast, multifractal analyses of motor series never exploited the temporal structure of scale exponents, and our work provides evidence supporting the feasibility of the MFMS-DFA also in these studies. In particular, we considered an experimental design where concurrent cognitive tasks during cycling limited the duration of the recordings to no more than 10 min. Such short recordings make particularly important the statistical consistency of the α(*q*,τ) estimates that characterize the MFMS-DFA method, thanks to its strategy of maximizing the overlapping between consecutive blocks. There are two other innovative solutions for the assessment of fractal analysis that can be applied using the MFMS-DFA and that the present work employs for the first time in a study on motor control. The first solution is the possibility of progressively shifting the order of the detrending polynomials fitting each block of data according to the block size *n*: The progressive increase from the first- to the second order by mixing the scale coefficients with a weighted average allowed us to efficiently remove second-order trends from the longer data blocks without introducing important polynomial overfitting in the shorter blocks (see also the Appendix A). The second solution is the use of the multifractal cumulative function for quantifying the degree of multifractality scale by scale: This index of multifractality is more stable compared to the traditional width of the Legendre spectrum, which often exhibits “zigzag shapes rather than the expected parabolic shape” on empirical series, as reported by other authors [9,25].

Our results highlighted the usefulness of a multiscale approach to multifractality also for motor time series because the MFMS-DFA identified alterations in the PRP fractal structure induced by the cognitive task, i.e., playing Tetris, separately at short (τ ≈ 16 s) and long (τ ≈ 64 s) scales (Figure 3e). The alterations occurring at short and long scales may have a different nature because the short-term alterations prevail in the less skilled participants compared to the other volunteers (Figure 6a–c), while long-term alterations appear with high statistical significance in the subgroup with the best Tetris scores in which, by contrast, short-term alterations are almost absent. A multifractal structure presenting different α coefficients at positive and negative moment orders *q* suggests that fractal processes with different amplitudes are acting simultaneously, being the influence of the fractal processes with lower amplitude amplified by *q* < 0 and decreased by *q* > 0 while the opposite is true for the fractal processes with higher amplitude. Thus, our results suggest that playing Tetris influences significantly the fractal processes with larger amplitude.

When Tetris was played in collaboration with another participant, the MFMS-DFA suggested a reorganization of the interactions among neural networks that may alter the fractal components with a lower amplitude (Figure 3d). The stratification by skill level provides further insights. While the multifractal structure of the PRP time series was again importantly altered in the less skilled subgroup (Figure 6d), we did not find significant alterations induced by the collaborative task in the subgroup with the best Tetris score, over the whole range of scales and for all the moment orders (Figure 6f). The present work, aimed at evaluating the applicability of the MFMS-DFA for assessing the fractal dynamics of human movements, is not designed to identify the mechanisms underlying the adaptations of brain motor centers during dual-task performance. However, our results appear consistent with the hypothesized phenomenon of dual-task interference, meaning that the performance of a specific task is impaired when the task is executed simultaneously with another task [15]. An explanation of dual-task interference is a limitation in the cognitive capacity of processing the flow of information. The latter has been experimentally associated with the limited recruitment of the neurons in the lateral prefrontal cortex: These neurons may be overloaded by the task-relevant information that increases proportionally with the demands of the concurrent tasks [26]. In our participants, we found significant changes in the MFMS-DFA of the PRP series at different scales and in all moment orders when cycling was performed while playing Tetris (Figure 3). These changes support the hypothesis that playing Tetris interferes with cycling. Actually, the dual-task performance is perceived as more demanding than the single task, according to the scores of the NASA-TLX questionnaire (Table 1). Interestingly, the NASA-TLX scores appear similarly high in CT and CTC compared to the single task in all the tertiles of Tetris best score. This indicates that the dual task was perceived as equally challenging by both more skilled and less skilled players. However, CTC is expected to be less cognitively demanding than CT, since the player can focus on one of the two actions of the Tetris game only: block rotation or block shift. Thus, we may hypothesize that in the less skilled players, the higher brain centers involved in the dual tasks were overloaded by the cognitive task of playing Tetris even when Tetris was played together with another participant. We may also hypothesize that in contrast the task of playing Tetris collaboratively was much less demanding for more skilled players. Thus, in the higher tertile of participants, the lower flow of information to be processed to play Tetris collaboratively allowed the higher brain centers to better elaborate the information required for the motor task of cycling. This would explain why in terms of the multifractal multiscale dynamics, the PRP series of the more skilled players did not differ substantially when cycling was executed as the single task or while playing Tetris collaboratively.

A final comment regards the assessment of multifractality by the MFMS-DFA approach. The multifractal cumulative function, α_CF_, was able to significantly identify the multifractality, which is expected in cyclic motor time series, over a wide range of scales. By describing the degree of multifractality as a function of τ, α_CF_ showed a stronger statistical significance at the larger scales (τ > 32 s, Figure 5), suggesting the superposition of fractal processes with long dynamics. In the comparison with the surrogate data, α_CF_ was also sufficiently sensitive to quantify more significant differences with the RP than the IAAFT surrogates, indicating that nonlinear dynamical components affect not only the Fourier phase but also the amplitude distribution of the PRP values [24].

In conclusion, the recently proposed MFMS-DFA provides statistically consistent estimates of the multifractal dynamics when applied on the relatively short time series typical of the studies on the interactions among the neural networks involved in motor control and cognitive processes. Its ability to quantify the multifractal structure separately over different ranges of scales may help to better identify the underlying mechanisms, with promising perspectives in the diagnosis of motor disorders and on the course monitoring of diseases affecting the neural networks involved in motor control.

### Limitations

Since the aim of the study was to evaluate the feasibility of the MFMS-DFA approach, the results should be considered carefully before proposing physiological interpretation or if they should be the reference in healthy controls for future comparison with diseased conditions. First, we may expect gender differences that require a larger population than the number of participants, 2/3 males and 1/3 females, enrolled in the present work. Second, our participants are healthy, young and middle-aged adults, and for future evaluations of the effects of specific diseases, likely to affect older ages, healthy volunteers properly matched by age with the patients are required.

## Figures and Tables

**Table 1:** Participants’ characteristics by tertiles of Tetris score.

	I Tertile	II Tertile	III Tertile
Females/Males	4/8	5/7	3/9
Age (yoa)	34 (15)	32 (13)	28 (9)
*NASA-Task Load Index of Total Workload*			
C	46.3 (27.8)	43.3 (21.2)	47.7 (11.3)
CT	64.7 (23.8)	60.3 (12.3)	66.7 (18.7)
CTC	65.3 (20.8)	67.0 (18.7)	63.3 (10.2)

Values as mean (standard deviation) for age, as median (interquartile range) for NASA-TLX questionnaire scores.

**Figure A1:**
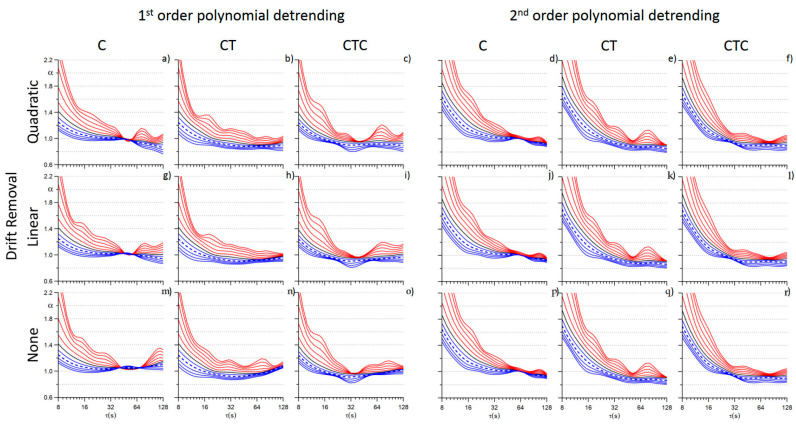
Scale coefficients after quadratic (**a**–**f**) or linear (**g**–**l**) drift removal or without drift removal (**m**–**r**) calculated with first- or second-order detrending in each of the three experimental conditions. Colors as in Figure 2.

**Figure A2:**
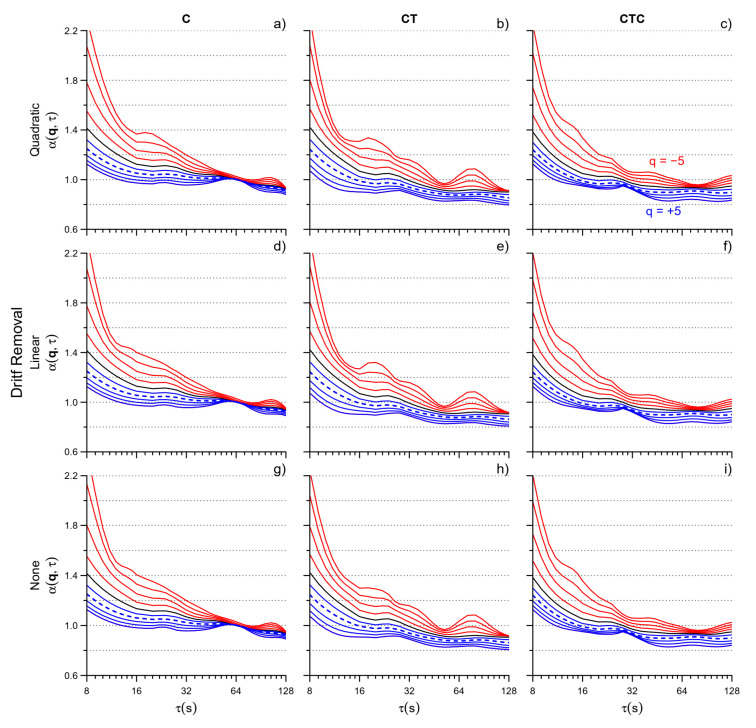
MFMS DFA coefficients estimated with a weighted average of the scale coefficients obtained by first- and second-order detrending: values calculated after quadratic (**a**–**c**) and linear (**d**–**f**) drift removal and without drift removal (**g**–**i**). Colors as in Figure 2.

## Data Availability

The data supporting the main findings of this study are available on reasonable request with access granted on justified request to researchers meeting the criteria for access to confidential data.

## Appendix A

Possibly present long-term drifts in the recordings may influence the DFA coefficients, and trend removal is generally recommended [27]. Drifts can be removed by subtracting linear or quadratic polynomials least-square fitting the series. However, to calculate *F_q_*(*n*) in Equation (1), the DFA already detrends each block of size of *n* samples by a polynomial fitting. In our approach, we used both first- and second-order detrending polynomials, which may have mitigated differently the effects of long-term drifts. Thus, to evaluate how importantly long-term drifts may have influenced our results, we calculated the MFMS DFA coefficients, α(*q*,τ), without drift removal and removing linear or quadratic drifts. This was carried out using both detrending polynomials of first and second order (Figure A1). The results show that when the second-order polynomial detrending is used within each block of size *n*, the α(*q*,τ) coefficients without or with drift removals are virtually identical. This means that long-term linear or quadratic drifts are automatically removed at all the scales by the DFA method itself which employs the second-order detrending.

We also calculated α(*q*,τ) coefficients using the first-order detrending to avoid overfitting effects. Figure A1 shows that in this case only the coefficients estimated at the larger scales (τ > 30 s) are influenced by the drift removal.

Our final estimate mixes the two detrending orders with a weighted average of the α(*q*,τ) coefficients after first- and second-order detrending. In the weighted average, estimates with first-order detrending prevail at the shorter scales (to remove the overfitting effect) but have no weight at the larger scales to fully detrend the larger blocks with a parabolic fitting. Thus, we do not expect any substantial influence of drift removal on our estimates. This is confirmed by Figure A2: The mixed coefficients, calculated either without or with drift removal, are virtually identical.
